# Exosome-transmitted circ_002136 promotes hepatocellular carcinoma progression by miR-19a-3p/RAB1A pathway

**DOI:** 10.1186/s12885-022-10367-z

**Published:** 2022-12-07

**Authors:** Peng Yuan, Jinhua Song, Fei Wang, Baoan Chen

**Affiliations:** 1 Department of Hematology and Oncology (Key Discipline of Jiangsu Medicine), Medical School, Zhongda Hospital, Southeast University, Dingjiaqiao No.87, Gulou District, 210009 Nanjing, Jiangsu Nanjing, People’s Republic of China; 2 Interventional Vascular Department, Jianhu People’s Hospital, Yancheng, Jiangsu China; 3 Hepatobiliary Center, The First Affiliated Hospital of Nanjing Medical University, Nanjing, Jiangsu China

**Keywords:** Hepatocellular carcinoma, Exosome, circ_002136, miR-19a-3p, RAB1A

## Abstract

**Background:**

Circular RNAs (circRNAs) are enriched in exosomes and are extremely stable. Exosome-mediated intercellular transfer of specific biologically active circRNA molecules can drive the transformation of the tumor microenvironment and accelerate or inhibit the local spread and multifocal growth of hepatocellular carcinoma (HCC). In this study, we explored in depth about the biological roles of HCC cell-derived exosomes and exosome-transported circRNAs on HCC in vivo and in vitro.

**Methods:**

Exosomes extracted from HCC cells (Huh7 and HA22T) were characterized using transmission electron microscopy, nanoparticle size tracer analysis, and western blotting. Exosomes were observed for endocytosis using fluorescent labeling. The effects of HCC cell-derived exosomes and the circ_002136 they carried on cell growth, metastasis and apoptosis were determined by CCK-8 assay, transwell assay, flow cytometry analysis and TUNEL staining, respectively. The expressions of circ_002136, miR-19a-3p and RAB1A were detected by quantitative RT-PCR (qRT-PCR). Targeted binding between miR-19a-3p and circ_002136 or RAB1A was predicted and verified by bioinformatics analysis, dual-luciferase reporter and RNA pull-down experiments. The in vivo effect of circ_002136 was determined by constructing a xenograft tumor model.

**Results:**

The findings revealed that Huh7 and HA22T exosomes conferred enhanced viability as well as invasive ability to recipient HCC cells. Circ_002136 was shown for the first time to be differentially upregulated in HCC tissues and cells and transferred by HCC cell-derived exosomes. More importantly, selective silencing of circ_002136 depleted the malignant biological behaviors of HCC exosome-activated Huh7 and HA22T cells. Depletion of circ_002136 in *vivo* effectively retarded the growth of HCC xenograft tumors. Furthermore, a well-established circ_002136 ceRNA regulatory network was constructed, namely circ_002136 blocked miR-19a-3p expression, elevated RAB1A expression activity and stimulated HCC development. Finally, high levels of circ_002136 or RAB1A, as well as low levels of miR-19a-3p, negatively affected HCC patient survival.

**Conclusion:**

The study on circ_002136 provides good data to support our insight into the mechanism of to-be-silenced circRNA as a therapeutic agent in the progression of HCC.

**Supplementary Information:**

The online version contains supplementary material available at 10.1186/s12885-022-10367-z.

## Introduction

Primary liver cancer (PLC) has developed into the 3rd most lethal tumor and 6th most common cancer worldwide, with more than 800,000 new cases and deaths worldwide each year [1]. Hepatocellular carcinoma (HCC), as the main pathological subtype of PLC, accounts for about 75-85% of the total number of cases [2]. HCC is one of the most difficult tumors to treat because of its insidious onset and ease of intrahepatic dissemination. The fact is that HCC is often in the progressive stage when diagnosed, with an extremely prognosis, and often dying within six months without treatment [3, 4]. Therefore, exploring the specific mechanisms of HCC growth in vivo and mastering new treatment strategies have become the sticking point to ameliorate the prognosis of patients with hepatocellular carcinoma.

Exosomes (Exo) are round or oval nanoscale vesicles with a diameter of 30–200 nm formed by the process of “endocytosis-fusion-exocytosis” of living cells, which can aggregate and deliver proteins, lipids and genetic materials [5, 6]. As carriers of information exchange between tumor cells, tumor cells and their surroundings, exosomes act a role of communication bridge in tumors by inducing exchange of oncogenic factors between adjacent or distant tumor cells through the transport and delivery of bioactive molecules [7], and are implicated in the diagnosis, metastasis, treatment, and even drug resistance of malignant tumors [8, 9]. It has been demonstrated that, compared to other cell types, tumor cells can actively release high abundance of exosomes into the extracellular environment or even spread to distant sites [10]. More interestingly, the aggressive tumor cells can use the “mobility” of exosomes to confer malignant characteristics to sensitive cells [11]. Thus, exosomes show great potential to modulate and alter the biological features of malignant tumors.

Circular RNAs (circRNAs) are single-stranded closed specific non-coding RNA molecules that are produced by a classical reverse splicing mechanism and can manage gene expression in a wide variety of biological cells [12]. The deviant expression of circRNAs in vivo can alter the normal growth, apoptosis and other processes of tumor cells, suggesting their ability to be used as an aid in tumor diagnosis and in surveilling tumor progression [13]. It has been shown that circRNA loaded in exosomes can continue to function after exosomes are taken up by neighboring cells [14]. Moreover, circRNAs that exist in a special ring-like closed structure are protected by the nano-phospholipid bilayer structure of exosomes, which prevents further removal, damage or degradation of the internal “cargo” and contributes to prolonging the circulating half-life of circRNAs [15]. Therefore, the diverse and specific circRNAs in exosomes make them sensitive biomarkers for tumor prediction, diagnosis and treatment. Human circ_002136 (hsa_circCDK11A_001) presented enriched level in malignant glioma cells and tissues [16]. More importantly, overproduced circ_002136 generated a promoting effect on glioma angiogenesis in vivo. This is the first evidence on the function and mechanism of circ_002136 in human cancer. However, so far, the regulatory network and downstream mechanisms of exosomal circ_002136 in HCC have not been reported.

In this study, we identified circ_002136 (hsa_circCDK11A_001) as an important exosomal circRNA in the malignant process of HCC and confirmed that HCC cell-secreted exosomes can propagate circ_002136 between cells exacerbating the aggressiveness and migration of cancer cells. Notably, bioinformatic analysis also confirmed endogenous competitive roles between circ_002136, miR-19a-3p and RAB1A. Specifically, circ_002136 was validated to negatively regulate miR-19a-3p to increase RAB1A expression, forming a feedback loop, which positively contributed to the growth of HCC tumors. This study reflects the need to assess the functional contribution of exosomal circRNAs to HCC and the interactions between exosome-associated tumor cells.

## Materials and methods

### HCC tissue samples and cell cultures

The 35 samples (HCC specimens and paired paracancerous tissues) for this study were collected from Jianhu People’s Hospital. Patients with HCC who had been treated or had other solid tumors were not included in this study. The collection and use of human tissues for the study was permitted and authorized by Ethical Review Committees of Jianhu People’s Hospital, and informed consent was obtained from all patients. Table 1 described the relationship between circ_002136 expression and clinical severity of HCC patients. We drew Kaplan-Meier survival curves according to the relative expression levels and cutoff values of circ_002136, miR-19a-3p and RAB1A. All experiments were performed in accordance with relevant guidelines and regulations and in compliance with the *Declaration of Helsinki.*

**Table 1:** Association between clinical features and hsa_circ_002136 expression of HCC patients

Clinicopathologic characteristics	*n*	hsa_circ_002136		
		**Low** **(n = 18)**	**High** **(n = 17)**	***p*** **-value**
Age (years)				0.6254
< 55	20	11	9	
≥ 55	15	7	8	
Sex				0.1267
Male	18	7	11	
Female	17	11	6	
Tumor size				0.0272*
<50 mm	21	14	7	
≥ 50 mm	14	4	10	
T stage				0.0105*
I–II	16	12	4	
III–IV	19	6	13	
Lymph node involvement				0.0284*
Absent	19	13	6	
Present	16	5	11	

The 2 different human HCC cell lines (Huh7, HA22T) chosen for this work were acquired from the ATCC (American Type Culture Collection, USA). The human hepatic epithelial cell line (THLE-3) was provided by the Cell Bank of Chinese Academy of Sciences (Shanghai, China). Huh7, HA22T cells were maintained in RPMI 1640 medium (Gibco, USA) and THLE-3 cells were grown in normal DMEM medium. The basal medium utilized was supplemented with 1% penicillin/streptomycin (Sigma, USA) and 10% FBS (Gibco, USA), followed by placing in an incubator (culture environment: 37 °C, 5% CO_2_).

### Isolation and identification of exosomes from culture media

Exosomes from Huh7, HA22T cells were isolated from vesicle depletion medium (20 ml). Briefly, HCC cells were stored in culture for 48 h, then the culture medium was harvested, followed by centrifuging at 1,000×g for 10 min, 3,000×g for 30 min, and 10,000×g for 60 min at 4 °C. After each isolation step, the supernatants were filtered through a 0.22 μm membrane (PVDF, Millipore, USA) to sequentially eliminate dead cells, cell debris, and dislodged vesicles. Finally, the supernatants were continued to be purified and centrifuged at 100,000×g for 4 h to harvest the exosome precipitate. The obtained purified exosome samples were re-suspended in PBS and stored at -80 °C for backup. Exosome morphology was collected using transmission electron microscopy (TEM). Exosome particle size data were acquired using nanoparticle tracking analysis (NTA) software (ZetaView 8.02.28). The levels of characteristic proteins CD63, CD9 and Hsp70 on the surface of exosomes were identified by western blot analysis. Notably, the PVDF membranes used for Western blot analysis were cut according to molecular weight before hybridization to the antibody.

### Exosome uptake and co-culture

Huh7 cell-derived exosomes were labeled with the green fluorescent labeling dye PKH67 (Sigma-Aldrich, Merck KGaA) according to the instructions. For cell processing, 2 × 10^5^ recipient cells were maintained with PKH67-labeled re-suspended exosomes for 24 h. DAPI was added for blue fluorescent labeling of Huh7 nuclei, and after washing, cell images were captured using a confocal laser scanning microscope (Nikon Eclipse Ti).

30 µg of exosomes provided from HCC cell lines were placed in 12-well plates, followed by the independent addition of 1 × 10^6^ Huh7 or HA22T cells. After 48 h incubation, CCK-8, Transwell, flow cytometry analysis and TUNEL staining of Huh7 or HA22T cells were performed.

### Cell transfection

To alter the expression of circ_002136, miR-19a-3p, and RAB1A, two small hairpin RNAs (si-circ_002136 1#, si-circ_002136 2#) targeting the linkage region of circ_002136 and its negative control (si-NC), miRNA mimic/inhibitor for over/low-expressing of miR-19a- 3p and its matching negative control plasmids (mimic nc and inhibitor nc), were obtained from Anhui General Biological Company (China). The RAB1A sequence was cloned into the empty pcDNA3.1 vector used to generate an overexpression plasmid for RAB1A, labeled as RAB1A (empty vector controls were marked as vector). Transfection of siRNA oligonucleotides or vectors was achieved in Huh7 or HA22T cells using Lipofectamine 3000 (Invitrogen, USA), according to the manufacturer’s instructions.

### Cell viability assay

Cell proliferation activity was assayed by Cell Counting Kit-8 (CCK-8) kit. 96-well plates were added with cell suspensions (5 × 10^3^ cells/well, 100 µL) and pre-incubated in RPMI-1640 medium for 24 h, respectively. Next, incubation was proceeded with or without the addition of HCC cell exosomes (20 µL, 0.1 µg/µL) for 24 h. All cells were mixed with 10 µL CCK-8 reagent for another 1 h. Finally, the optical density (OD) values of each well were quantified at 450 nm using an enzyme marker (BioTek Instruments).

### Cell apoptosis analysis

The percentage of apoptotic cells was analyzed using Annexin V PE/7-AAD Apoptosis Detection Kit (Nanjing Jiancheng Bioengineering Institute, China). Huh7 and HA22T cells were inoculated on 6-well plates at a high density of 5 × 10^5^ /well. The cells were collected by centrifugation and washed with PBS to prepare cell suspensions following exosome treatment or not. The cell suspensions were stained with Annexin V/PE and 7-AAD for 15 min in a dark environment. Finally, stained positive samples were evaluated using flow cytometry (BD Biosciences, USA).

### TUNEL staining

The TUNEL kit (Thermo Fisher) was performed to identify apoptosis in exosome-treated/untreated HCC cells. After the prescribed treatment according to the manufacturer’s protocol, Huh7 or HA22T cells were fixed by incubation with 4% (w/v) paraformaldehyde for 15 min at 4 °C. Next, the desired cells were sequentially incubated with TUNEL reaction liquid and DAPI for staining. Where DAPI was utilized to stain the nucleus (blue), and TUNEL staining (green) was applied to label apoptotic cells. Finally, fluorescence microscopy (CX31-P, Olympus) was employed to determine the number of TUNEL-positive cells after magnified imaging, and the degree of apoptosis was evaluated.

### Transwell assay

Migration and invasion of Huh7 or HA22T cells were assessed using 24-well 8 μm Transwell chambers (Millipore, USA). For migration experiments, the cell concentration was adjusted to 2 × 10^4^ cells/200 µL and then the cell solutions (Huh7 cells, Huh7 cells and exosomes, HA22T cells, HA22T cells and exosomes) were inoculated in the Transwell upper chamber. 800 µL of chemo-attractant (RPMI1640 medium + 10% FBS) was added to the lower chamber. After maintenance for 24 h, cotton swabs were wiped off the unmigrated cells in the upper chamber, the surface-migrated cells were fixed with methanol at room temperature for 10 min, stained with 0.1% crystal violet for 5 min, and five random fields were selected for observation and counting under an Olympus inverted microscope (magnification ×200). The steps of cell invasion analysis were processed in a similar manner to migration analysis except that HCC cells needed to be introduced into the upper chamber pretreated with Matrigel solution (BD Biosciences).

### Dual luciferase reporter gene assay

The wild-type or mutant circ_002136 sequence containing the miR-19a-3p binding site, the 3’-UTR wild-type or mutant sequence of RAB1A mRNA were inserted into the p-mir-GLO luciferase vectors (Promega, Madison, WI, USA) to obtain the desired luciferase reporter vectors (WT-circ_002136, MUT-circ_002136, WT-RAB1A and MUT-RAB1A). HCC cells were cotransfected with the above luciferase reporter vectors in 96-well plates, together with miR-19a-3p mimics or miR-NC. 48 h later, dual luciferase reporter gene assay system (Promega, USA) was constructed to quantify the corresponding luciferase activity.

### Biotin miRNA pull-down assay

Purified miR-19a-3p was biotin-labeled as biotin-miR-19a-3p (Sangon Biotech) and the corresponding negative control probe (biotin-NC) was obtained. The biotinylated miR-19a-3p, negative control (biotin-NC) and positive control (input) were co-incubated with HCC cell lysate supernatant at room temperature, followed by the addition of Dynabeads M-280 streptavidin magnetic beads (Thermo Scientific) at 25 °C to obtain the probe-bead complexes. And finally, the beads-bound RNA complexes were pulled down and the bound RNA was quantified by qRT-PCR.

### qRT-PCR

Isolation of total RNA from the obtained HCC tissues and target group of HCC cells was completed with the help of TRIzol (Invitrogen, USA) kit. 500 ng of target RNA samples (circ_002136, miR-19a-3p, RAB1A) were directly reverse transcribed to cDNA by operating PrimeScript™ RT Master Mix (Takara). 7500 Fast RT-PCR system (Applied Biosystems) and SYBR ® Premix Ex Taq™ (Takara) were carried out to process qRT-PCR assay. U6 was acted as an internal control for miR-19a-3p, and β-Actin was applied as internal controls for RAB1A and circ_002136. The relative expression levels of the target genes were calculated by the 2^–∆∆Ct^ method. The primer sequences of the specific genes used were:

circ_002136-F: 5’-CCGCATGGAGATCACAATAA − 3’.

circ_002136-R: 5’-TTTTCTTTCCGAGACATTTGC − 3’.

miR-19a-3p-F: 5’-TGCGGUUGCAAAUCUAUGCAAAACUG-3’.

miR-19a-3p-R: 5’-CCAGTGCAGGGTCCGAGGT-3’.

RAB1A-F: AGATTAAAAAGCGAATGGGTCCC

RAB1A-R: GCTTGACTGGAGTGCTCTCTGAAT

U6-F: 5'-GAGGCACAGCGGAACG-3'

U6-R: 5'-CTACCACATAGTCCAGG-3'

β-Actin-F: 5'-CTCCATCCTGGCCTCGCTGT-3'

β-Actin-R: 5'-GCTGTCACCTTCACCGTTCC-3'

### Functional verification of circ_002136 ***in vivo***

All animal experiments were performed in strict accordance with institutional guidelines and were approved by the Jianhu People’s Hospital Ethics Committee. Male C57Bl/6 nude mice, 6 ~ 8 weeks old, weighing 18–20 g, were provided by SLAC Laboratory Animals Co., Ltd. (Shanghai, China). Mice to be operated on were randomly divided into two groups (adenovirus (Ad)-sh-circ_002136 group and Ad-sh-NC group) of three mice each for subsequent studies. sh-NC and circ_002136-specific short hairpin RNA (sh-circ_002136) were both inserted by Sangon Biotech (Shanghai, China) into adenovirus (Ad) and transduced into HCC cells. HCC cells transfected with adenoviral vectors were re-suspended in PBS. Cells in 100 µL PBS were then subcutaneously inoculated into C57Bl/6 mice at a viable density of 1 × 10^6^ cells/ml. Mice were measured for tumor volume and size every seven days until 35 days post-inoculation, calculated as 0.5 × (length × width^2)^. After 35 days, mice were executed and tumors were excised, weighed and photographed.

### Statistical analysis

The used experiments were performed at least three times independently and the data obtained are expressed as mean ± SD. Student’s t-test or One-way ANOVA was subjected to calculate statistical differences between the two or multiple groups. The statistical analyses of gene data were operated via SPSS 20.0 statistical software (Chicago, IL, USA) and graphed in Prism software (GraphPad, Version 8.1.1, CA, USA). P-value of less than 0.05 was considered as the criterion for statistical significance.

## Results

### HCC cell-derived exosomes exacerbate the malignant process of HCC ***in vivo***

**Fig. 1:**
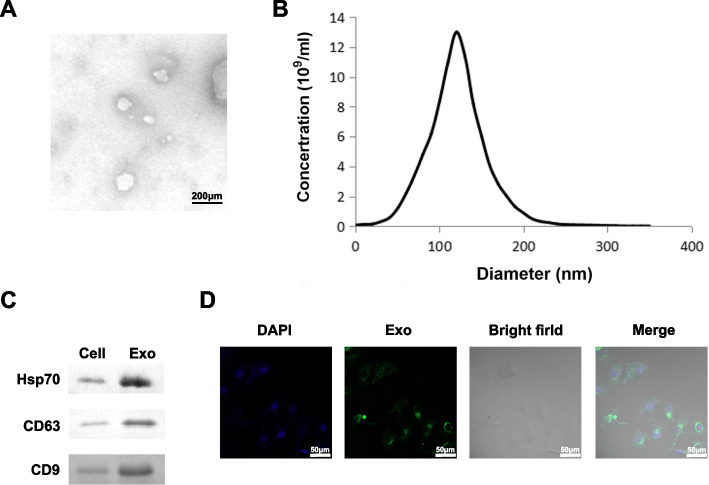
Characteristic of HCC-derived exosomes. **A**, **B** TEM and NTA were applied to identify and measure the morphology and particle size range of exosomes observed from HCC cell culture media, respectively. Scale bars, 200 nm. NTA analysis showed that the existing exosomes ranged in diameter from 100 to 200 nm. **C** Identification of the presence of exosomal marker proteins CD63, CD9 and Hsp70 by western blot analysis. HCC cell lysates were served as positive control. The membrane was cropped according to the molecular weight of the protein before the incubation with primary antibodies. GAPDH was used as a control. Full-length blots/gels are presented in Additional file 2. **D** Green fluorescent signal of internalized exosomes in HCC cells was detected by confocal laser scanning microscopy. Scale bars, 50 μm

Tumor cells can interfere with the behavior of receptor cells by delivering and exchanging exosomes inclusions with surrounding cells. Therefore, we focused on exploring exosome-based mechanisms for HCC progression. First, we obtained exosomes from the culture supernatant of HCC high metastatic potential cell lines (Huh7, HA22T) by high-speed centrifugation. Under TEM, HCC cell line-derived exosomes exhibited a typical round appearance, a transparent and intact lipid membrane appeared around the exosomes (Fig. 1A). NTA results demonstrated that the peak diameters of these round particles were uniformly distributed in the range of 100–200 nm (Fig. 1B), which was consistent with the current common exosome size. CD63, CD9 and Hsp70 were abundant in the isolated particles compared to cell lysates (Fig. 1C and Fig. S2), further indicating their identity as exosomes. Exosome uptake by HCC receptor cells was identified with the help of PKH67 dye. As expected, confocal microscopy captured images showed the appearance of PKH67 green fluorescent signal in the perinuclear region of HCC cells, suggesting successful internalization of exosomes by receptor cells (Fig. 1D).

**Fig. 2:**
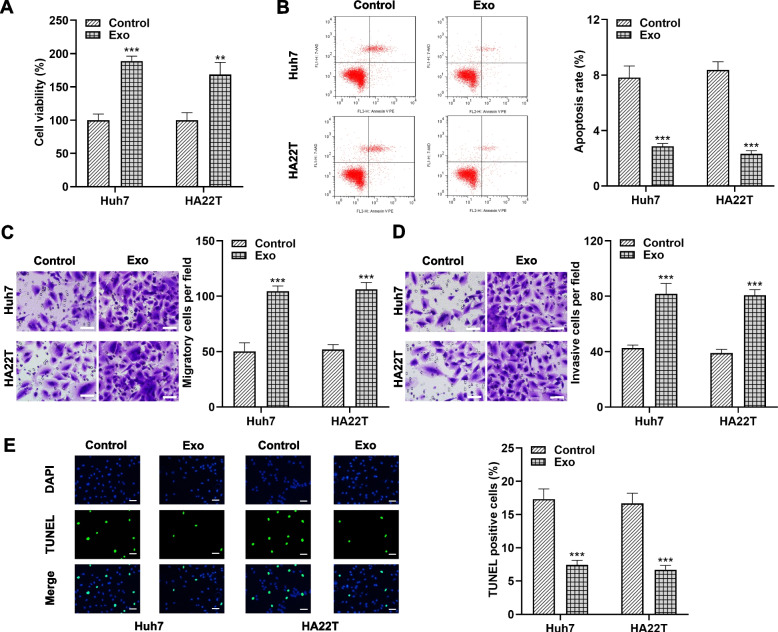
Hepatocellular carcinoma-derived exosomes induce tumor cell growth, migration and invasion ***in vitro***. **A** After 24 h of treatment with or without HCC cell-derived exosomes, Huh7 and HA22T cell activities were examined separately by CCK-8 assay to characterize the altered growth activity. **B** Annexin V/PE and 7-AAD staining and flow cytometry were performed on Huh7 and HA22T cells in the presence or absence of exosomes. **C**, **D** Transwell analysis was performed on Huh7 and HA22T cells after co-incubation with or without HCC cell-derived exosomes for 24 h to assess their migratory (**C**) and invasive (**D**) abilities. Scale bar, 50 μm. **E** TUNEL analysis illustrated the effect of exosome treatment on HCC cell apoptosis. Scale bar, 50 μm. *** *P* < 0.001. *** *P* < 0.001 indicates compared to control group

Exosome regulation of HCC cell metastasis and invasion has been demonstrated [17], and we found that exosomes conferred higher viability to Huh7 and HA22T cells by establishing a co-culture system (Fig. 2A). Flow cytometry analysis confirmed that the apoptosis rate of HCC cells exposed to exosomes was lower than that of control cells (Fig. 2B). In addition, Transwell invasion and migration assays demonstrated that the additional addition of exosomes resulted in aggravated migration and invasion of Huh7 and HA22T cells, as evidenced by an increase in the number of migrating and invading cells (Fig. 2C and 2D). TUNEL staining results also showed that exosomes treatment reduced the proportion of TUNEL-positive cells and inhibited apoptosis of HCC tumor cells (Fig. 2E). In conclusion, our data emphasize that exosomes take up a crucial part in mediating the malignant biological behaviors of HCC.

### Exosomal circ_002136 is highly expressed in HCC and predicts poor survival of HCC patients

**Fig. 3:**
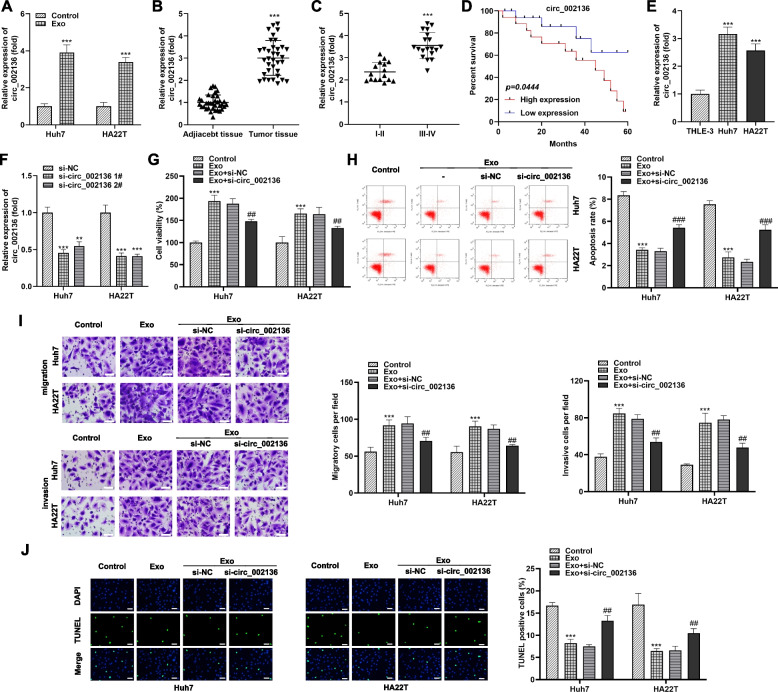
Promotion of malignant behavior of HCC cells by exosomes is dependent on the degree of circ_002136 enrichment. **A** Expression of circ_002136 in Huh7 and HA22T cells exposed to exosomes was assessed by qRT-PCR, separately. **B** qRT-PCR was employed to measure the levels of circ_002136 in HCC samples (*n* = 35) and paired para-cancer normal samples (*n* = 35). (Terminal deoxynucleotidyl transferase (TdT)-mediated dUTP nick-end labeling) The levels of circ_002136 were lower in stage I-II HCC tissues than in stage III-IV specimens. **D** Survival curves of HCC patients in the circ_002136 high expression group and circ_002136 low expression group were conducted to assess prognostic significance. **E** qRT-PCR was subjected to detect differences in circ_002136 expression in diverse cell lines (THLE-3, Huh7, HA22T). **F** Compared with si-NC, qRT-PCR was applied to evaluate the circ_002136 levels in Huh7 and HA22T cells transfected with si-circ_002136 1# and si-circ_002136 2#. (**G**-**J**) Changes in cell viability, apoptotic capacity, migration and invasion capacities of Huh7 and HA22T cells treated with Exo, Exo + si-NC, Exo + si-circ_002136 were characterized by CCK-8 (G), flow cytometry (**H**), Transwell (I), and TUNEL staining (**J**) respectively. Scale bar, 50 μm. ** *P* < 0.01, *** *P* < 0.001, ^##^ *P* < 0.01,^###^ *P* < 0.001. ** *P* < 0.01 indicates compared with si-NC group; *** *P* < 0.001 indicates compared with control group, adjacent tissue group, stage I-II group, THLE-3 cell group, si-NC group; ^##^ *P* < 0.01 and ^###^ *P* < 0.001 indicate compared with Exo + si-NC group

Exosomes trigger malignant transformation of disease by delivering circRNA. To determine which circRNAs were loaded in exosomes and served as the primary malignant message, we selected 10 exosomal circRNAs (circ_0091579, circ_0000847, circ_0008537, circ_0013290, circ_0001313, circ_0005046, circ_0001791, circ_0004913, circ_002136, circ_0124554) through a systematic literature study, and their levels in two cell lines (Huh7 and HA22T) treated with exosomes were verified and screened by qRT-PCR. The results showed that three of the ten circRNAs (circ_002136, circ_0008537, circ_0005046) were significantly elevated in exosome-exposed HCC cells (Figures S1A and S1B). More importantly, compared to circ_0008537 and circ_0005046, circ_002136 displayed the most significantly increased level. These findings suggested that circ_002136 was delivered by HCC cell-derived exosomes and motivated us to continue to explore the main mechanism of circ_002136 in hepatocellular carcinoma. Therefore, based on exosomes, we again examined the levels of HCC-associated circ_002136 in highly metastatic Huh7 and HA22T cells. qRT-PCR results confirmed that Huh7 and HA22T cells treated with exosomes possessed more abundant levels of circ_002136 compared with the original cells (Fig. 3A). Subsequently, we examined the variation of circ_002136 levels in the obtained tumor samples. As shown in Fig. 3B, the abundance of circ_002136 was higher in HCC tumor tissues (*n* = 35) than in paired normal tissues (*n* = 35). In addition, patients classified as stage I-II HCC had lower levels of exosomal circ_002136 than patients with stage III-IV HCC (Fig. 3C). Based on the above results, to evaluate the clinical value of circ_002136, we next analyzed the association between the expression level of circ_002136 and clinicopathological parameters of HCC patients. First, referring to the critical value (≥ 3.037), HCC patients were divided into circ_002136 high expression group (*n* = 17) and circ_002136 low expression group (*n* = 18). Subsequently, the clinical manifestations of the subject patients were compared. As shown in Table 1, patients in the high circ_002136 group were more prone to have tumors with a diameter of ≥ 50 mm (*P* = 0.0272), and were more likely to have advanced tumors (T) (*P* = 0.0105) and lymph node involvement (*P* = 0.0284). However, no correlation was found between circ_002136 levels and other clinicopathological factors, such as age (*P* = 0.6254), gender (*P* = 0.1267). Furthermore, higher survival was also observed in HCC patients with lower levels of circ_002136 compared to those with higher levels (Fig. 3D). Likewise, we observed that the abundance of circ_002136 was similarly higher in Huh7 and HA22T cells than in THLE-3 (Fig. 3E). These results hinted that higher circ_002136 level might predict poor survival outcomes in HCC patients.

### Silencing of exosomal circ_002136 hinders the malignant processes of HCC cells

**Fig. 4:**
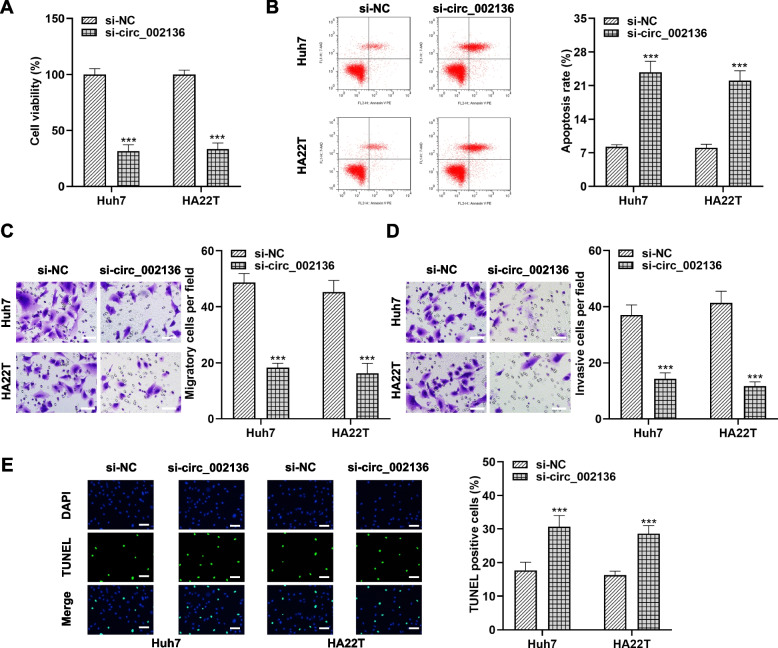
Lower expression of circ_002136 resists the malignant behaviors of HCC cells. **A**-**E** Changes in viability (**A**), apoptotic capacity (**B**, **E**), migration (**C**) and invasion (**D**) capacities of Huh7 and HA22T cells were characterized by CCK-8, flow cytometry, TUNEL staining and Transwell after transfection with si-NC or s-circ_002136. Scale bar, 50 μm. *** *P* < 0.001. *** *P* < 0.001 indicates compared with si-NC group

It is not difficult to derive from the above results that the strengthened expression of exosomal circ_002136 may contribute to the maintenance of high viability of HCC cells. And exosomes might propagate circ_002136-mediated malignant features in this process. Considering the high expression of circ_002136 in HCC, we tried to perform loss-of-function analysis in Huh7 and HA22T cells. As expected, HCC cells manifested lower level of circ_002136 after transfection with both si-circ_002136 1# and si-circ_002136 2#, indicating successful knockdown (Fig. 3 F). Upon exosome stimulation, Huh7 and HA22T cells were greatly activated in proliferation and migration, followed by a decrease in apoptotic, an effect that could be partially rescued by si-circ_002136 (Fig. 3G-J). Our data implied that exosomes depended on maintenance of circ_002136 level to enhance the malignant biological behavior of HCC in vitro, while blocking intracellular circ_002136 expression counteracted the above effects. Further, we found that the proliferation, migration and invasion of HCC cells loaded with si-circ_002136 or si-NC alone were similarly restrained, while apoptotic activity was substantially raised (Fig. 4A-4E). Besides, the proliferative activity of HCC cells loaded with si-circ_0008537 or si-circ_0005046 was not significantly changed (Fig. S1C and S1D), which further confirmed the importance of focusing on circ_002136 in this study. Briefly, according to our results, the reduction of exosomal circ_002136 had an inhibitory effect on the proliferation and migration of HCC cells.

### Circ_002136 blocks miR-19a-3p in HCC cells

**Fig. 5:**
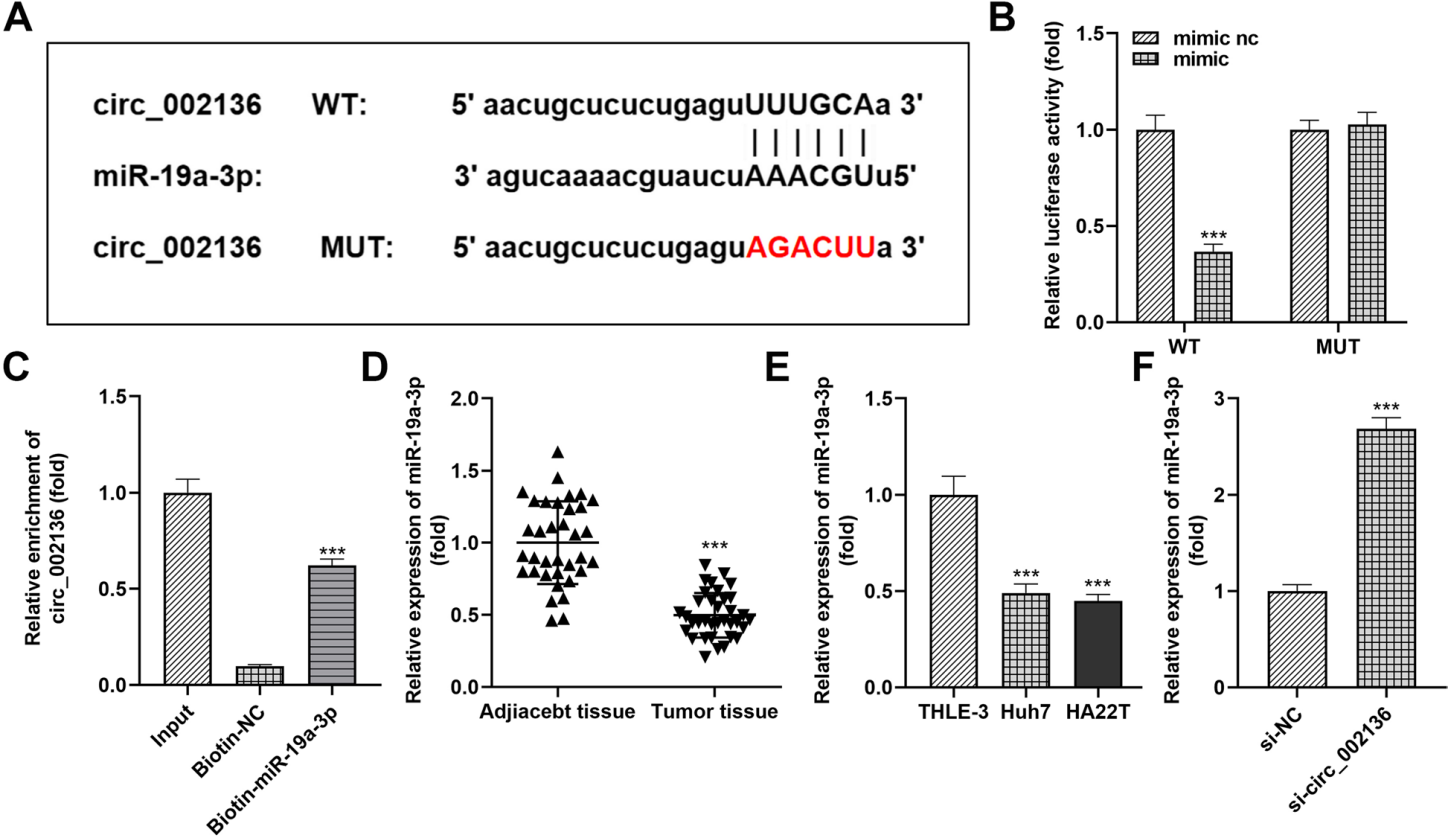
Circ_002136 functions as a miRNA sponge for miR-19a-3p in HCC. **A** MiR-19a-3p seed sequence and predicted target sites of wild-type or mutant circ_002136. **B** Analysis of luciferase activity in HCC cells co-transfected with miR-19a-3p mimic, mimic nc and luciferase reporter vector containing wild-type (WT) or mutant (MUT) circ_002136 sequences. **C** RNA pull-down assays were performed in HCC cells to confirm the direct binding of miR-19a-3p to circ_002136. **D** qRT-PCR was carried out to detect differential expression of miR-19a-3p in 35 pairs of samples (HCC tissues and paired non-tumor tissues). **E** qRT-PCR was performed to examine the differential expression of miR-19a-3p in several cell lines (THLE-3, Huh7, HA22T). **F** Introduction of si-circ_002136 in HCC cells increased the relative expression level of miR-19a-3p. *** *P* < 0.001. *** *P* < 0.001 indicates compared with mimic nc group, Input group, adjacent tissue group, THLE-3 group, and si-NC group

The ceRNA activity of circ_002136 has been proved in previous reports [16]. To explore and illustrate more clearly molecular mechanism of circ_002136 in HCC, we used StarBase (http://starbase.sysu.edu.cn/) to search for downstream target miRNAs of circ_002136. Finally, miR-19a-3p was locked as the target of circ_002136 (Fig. 5A). By constructing a dual luciferase reporter gene detection system, we found that the luciferase reporter plasmid (WT circ_002136) containing the miRNA predicted binding sites (wild type) induced a burst of intracellular luciferase activity after co-transfection with miR-19a-3p mimic. The mutant plasmid (MUT circ_002136) did not cause this change (Fig. 5B). Secondly, the results of RNA pull-down analysis also displayed that biotin-labeled miR-19a-3p could capture circ_002136 in large amounts within HCC cells (Fig. 5C). More interestingly, miR-19a-3p showed an apparently low expression trend in multiple HCC tissues and typical hepatocellular carcinoma cells, relative to circ_002136 expression pattern (Fig. 5D and E). Induction of circ_002136 reduction in cells increased the relative level of miR-19a-3p (Fig. 5F). The results obtained from the above experiments imply that circ_002136 can act on miR-19a-3p through special complementary seed regions, the two are mutually targeted for regulation.

### Inhibition of miR-19a-3p impairs the anti-cancer effects of circ_002136 silencing

**Fig. 6:**
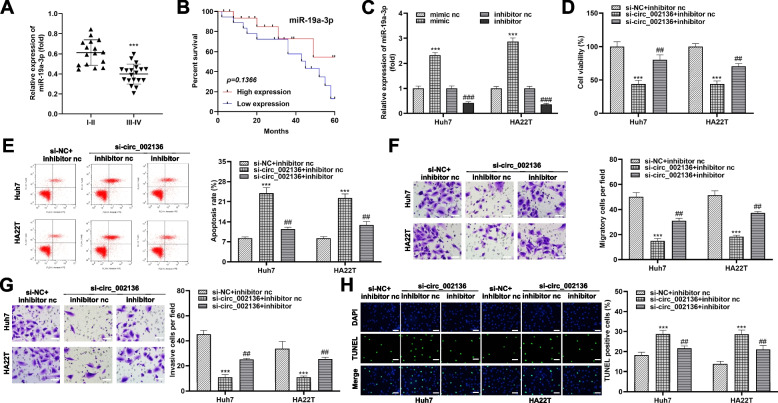
MiR-19a-3p inhibition impairs the restriction of HCC progression by si-circ_002136 ***in vitro***. **A** qRT-PCR was used to assess the relative expression of miR-19a-3p in specimens with different clinical stages of HCC. **B** Kaplan-Meier survival analysis was applied to determine the overall survival of the miR-19a-3p high and low expression groups. **C** Changes of intracellular miR-19a-3p in HA22T and Huh7 cells after treatment with miR-19a-3p mimic (mimic), mimic nc, miR-19a-3p inhibitor (inhibitor), or inhibitor nc (left panel). **D** HA22T and Huh7 cells were transfected with si-NC + inhibitor nc, si-circ_002136 + inhibitor nc, and si-circ_002136 + inhibitor, respectively, and CCK-8 was used to measure the changes of cell viability in different groups (right panel). **E** Flow cytometry experiment was performed to determine the changes of apoptotic activity in si-NC + inhibitor nc group, si-circ_002136 + inhibitor nc group, and si-circ_002136 + inhibitor group. **F**, **G** Transwell experiments were performed to characterize the cell migration (**F**) and invasion (**G**) abilities of si-NC + inhibitor nc group, si-circ_002136 + inhibitor nc group, and si-circ_002136 + inhibitor group. Scale bar, 50 μm. **H** TUNEL staining revealed the apoptotic capacity of HCC cells under different conditions. Scale bar, 50 μm. TUNEL positive cell ratio was also demonstrated (right panel). *** *P* < 0.001, ^##^ *P* < 0.01, ^###^ *P* < 0.001. *** *P* < 0.001 indicates compared with mimic nc group, stage I-II group and si-NC + inhibitor nc; ^##^ *P* < 0.01 indicates compared with si-circ_002136 + inhibitor nc group; ^###^ *P* < 0.001 indicates compared with the inhibitor nc group

We further investigated the possibility of miR-19a-3p involvement in HCC progression. In contrast to exosomal circ_002136, miR-19a-3p was expressed higher in HCC patients with stage I-II tumors than in patients with stage III-IV tumors (Fig. 6A). We also found that HCC patients with high levels of miR-19a-3p possessed more promising survival rates compared to those with low levels of miR-19a-3p (Fig. 6B). These findings suggested that miR-19a-3p was another important player in the progression of HCC. Therefore, we conjectured that circ_002136/miR-19a-3p could alter the proliferation and metastatic of HCC. Subsequently, we established miR-19a-3p inhibition and overexpression in HCC cells. MiR-19a-3p inhibitors and mimics successfully transformed the relative levels of miR-19a-3p upon introduction into HCC cells (Fig. 6C). Next, we designed rescue experiments using si-circ_002136 in Huh7 and HA22T cells, respectively. Data from CCK-8 assay showed that the proliferative activity of HCC cells decreased in the si-circ_002136 + inhibitor nc group compared to the si-NC + inhibitor nc group, also in response to the marked increase of apoptosis cells. Using the si-circ_002136 + inhibitor nc group as a control, the viability of HCC cells in the si-circ_002136 + inhibitor group was significantly increased, and correspondingly, the apoptotic activity was also decreased (Fig. 6D and E H). As indicated by the above results that the inhibition of miR-19a-3p successfully interfered with the hindering effect of silencing circ_002136 on malignant behaviors of HCC. In addition, changes in cell migration and invasion were consistent with the trend in cell viability (Fig. 6F and 6G).

### RAB1A expression was regulated by miR-19a-3p in HCC

**Fig. 7:**
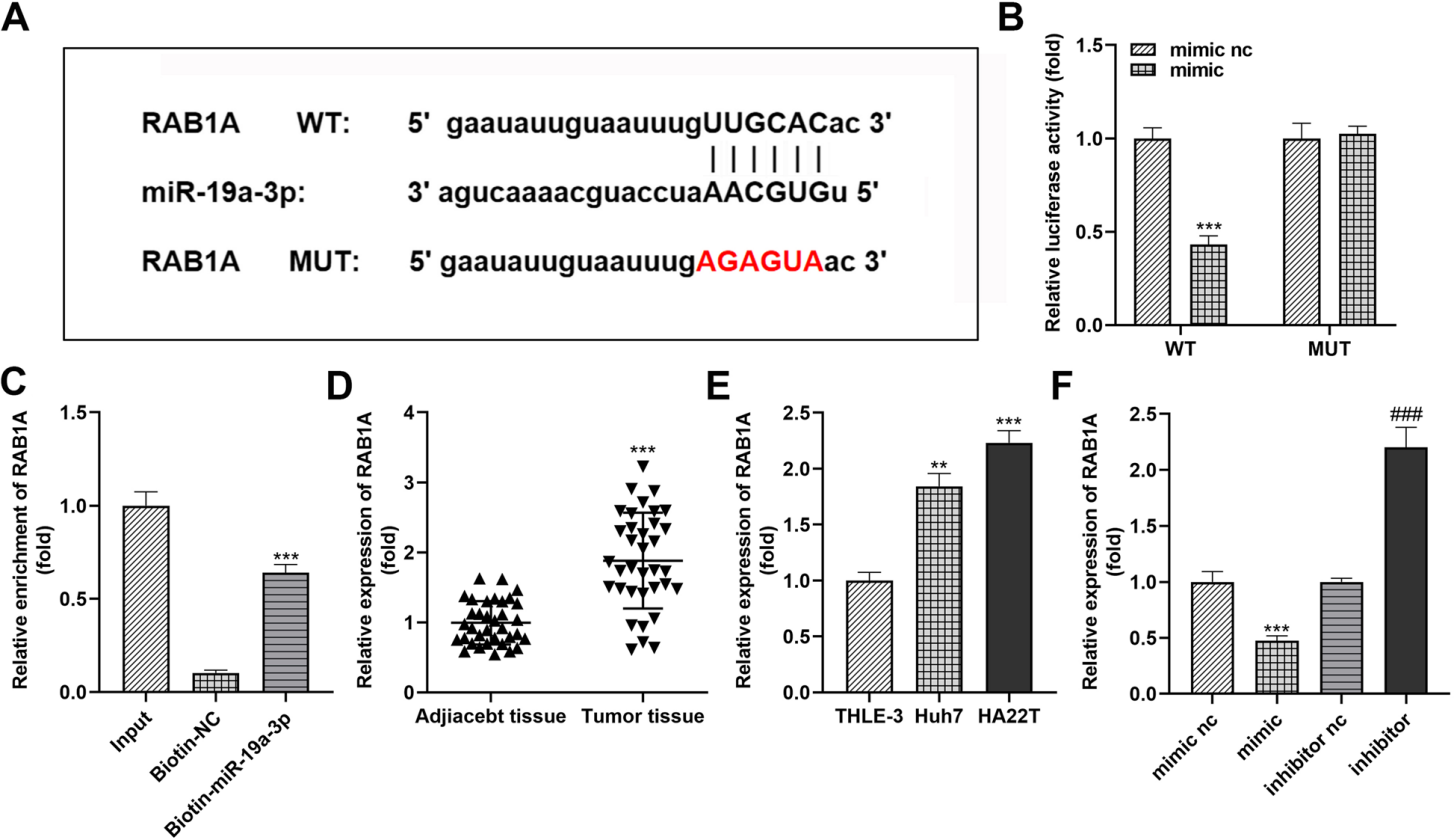
RAB1A is stably highly expressed in HCC and is a downstream target molecule of miR-19a-3p. **A** Binding sequence of miR-19a-3p to wild-type RAB1A 3’-UTR. **B** Analysis of luciferase activity in HCC cells co-transfected with miR-19a-3p mimic or mimic nc and luciferase reporter vectors containing wild-type (WT) or mutant (MUT) RAB1A sequences. **C** RNA pull-down assay was performed in HCC cells to confirm the direct binding of miR-19a-3p to RAB1A. **D** qRT-PCR was applied to detect differential expression of RAB1A in 35 pairs of samples (HCC tissues and paired non-tumor tissues). **E** qRT-PCR was performed to detect the differential expression of RAB1A in several cell lines (THLE-3, Huh7, HA22T). **F** The relative expression levels of RAB1A were differed after transfection of miR-19a-3p mimic (mimic), mimic nc, miR-19a-3p inhibitor (inhibitor) or inhibitor nc in HCC cells. ** *P* < 0.01, *** *P* < 0.001, ^###^ *P* < 0.001. ** *P* < 0.01 indicates compared with THLE-3 group; *** *P* < 0.001 indicates compared with mimic nc group, Input group, adjacent tissue group, THLE-3 group; ^###^ *P* < 0.001 indicates compared with inhibitor nc group

MicroRNAs can actively restrict the translation of target mRNAs and restrain their post-transcriptional expression [18]. To refine the regulatory pathway of circ_002136 and also to identify the downstream factors of miR-19a-3p, we applied the TargetScan (http://www.targetscan.org/vert_72/) database to predict the potential candidate target genes of miR-19a-3p. Based on the screening results, RAB1A was identified as a target gene for miR-19a-3p (Fig. 7A). The luciferase activity assay revealed that miR-19a-3p mimics resulted in sharply diminished luciferase activity in a reporter plasmid with a binding site sequence to miR-19a-3p (WT RAB1A) but not a reporter plasmid containing a RAB1A mutant sequence (MUT RAB1A) (Fig. 7B). RNA pull-down assays also evidenced that biotin-miR-19a-3p could couple RAB1A mRNA (Fig. 7C). In addition, RAB1A mRNA appeared high abundance expression in both HCC tumor tissues and cells, with an expression trend opposite to miR-19a-3p (Fig. 7D and E). As expected, intracellular RAB1A mRNA transcript levels were elevated or decreased upon transfection with miR-19a-3p mimics or inhibitors, respectively (Fig. 7F). These findings imply that RAB1A is a target of miR-19a-3p in HCC and that RAB1A activation is dependent on miR-19a-3p.

### RAB1A reverses the anti-tumor effect of miR-19a-3p ***in vitro***

**Fig. 8:**
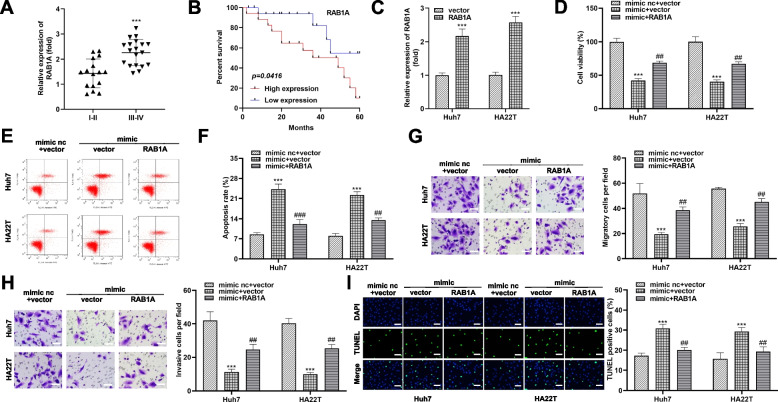
Higher RAB1A expression depletes miR-19a-3p-mediated suppression of HCC cells. **A** qRT-PCR was performed to analyze the relative levels of RAB1A in the obtained stage I-II and III-IV HCC tissue specimens. **B** Kaplan-Meier survival analysis was performed for HCC patients according to the levels of RAB1A. **C** Relative intracellular RAB1A mRNA levels in Huh7 and HA22T cells after treatment with RAB1A overexpression vector (RAB1A). **D** CCK-8 assays were operated to determine the changes of HCC cell viability after transfection with mimic nc + vector, mimic + vector, and mimic + RAB1A. **E**, **F** Apoptosis rates of Huh7 and HA22T cells were detected by flow cytometry. Data obtained by flow cytometry (left, E) and data analysis images (right, F) are shown. **G**, **H** Transwell assay was manipulated to assess cell migration and invasion abilities. The number of migrating and invasive cells (right) was calculated and the landmark images obtained by transwell (left) are shown. Scale bar, 50 μm. **I** Images represented TUNEL staining of HCC cells after transfection with mimic nc + vector, mimic + vector, and mimic + RAB1A. TUNEL positive cell ratio was also demonstrated (right panel). Scale bar, 50 μm. *** *P* < 0.001, ^##^ *P* < 0.01, ^###^ *P* < 0.001. *** *P* < 0.001 indicates compared to vector group and mimic nc + vector; ^##^ *P* < 0.01 and ^###^ *P* < 0.001 indicate compared to mimic + vector group

Consistently, we measured RAB1A expression levels in different HCC specimens. Interestingly, more abundant RAB1A levels were detected in early (stage I-II) HCC tissues, relative to advanced (stage III-IV) HCC tissues (Fig. 8A). In addition, poorer survival rate was also observed in HCC patients with higher RAB1A level (Fig. 8B). To analyze whether miR-19a-3p needs to function with the help of RAB1A in HCC, we designed and used an overexpression vector for RAB1A (denoted as: RAB1A), and subsequently, mimic nc + vector, miR-19a-3p mimic + vector and miR-19a-3p mimic + RAB1A were constructed to transfect HA22T and Huh7 cells. As shown in Fig. 8C, the overexpression vector effectively increased the level of RAB1A in both cell lines. Furthermore, HA22T and Huh7 cells manifested reduced cell viability, migration and invasiveness in the presence of high abundance of miR-19a-3p, corresponding to an elevation in apoptotic activity (Fig. 8D and I). However, the additional addition of RAB1A overexpression vector weakened the effect of miR-19a-3p (Fig. 8D and I). According to our results, miR-19a-3p blocked RAB1A to inhibit the malignant process of HCC.

### Silencing circ_002136 blocks HCC tumor growth ***in vivo***

**Fig. 9:**
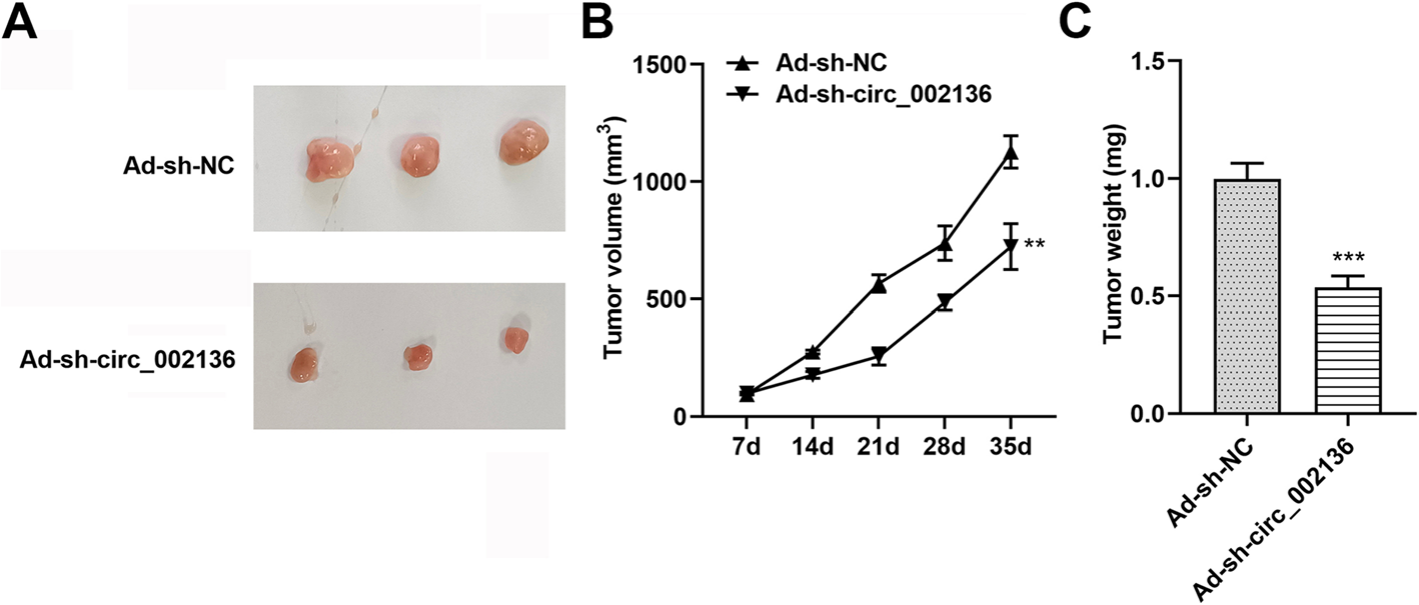
Circ_002136 low expression slows down HCC growth ***in vivo***. HCC cells stably expressing circ_002136 (labeled as Ad-sh-NC group and Ad-sh-circ_002136 group) were subcutaneously inoculated into C57Bl/6 nude mice to construct xenograft models. **A**, **B** Representative images of tumors formed in nude mice and analysis of tumor volumes (3 per group). **C** Ad-sh-circ_002136 group with reduced xenograft weight (3 per group). ** *P* < 0.01 and *** *P* < 0.001 indicate compared to the Ad-sh-NC group

To investigate the effect of hsa_circ_002136 on tumorigenesis in vivo, we injected HCC cells infected with control adenovirus (Ad-sh-NC) and adenovirus harboring sh-circ_002136 (Ad-sh-circ_002136) into the left side of C57BL/6 nude mice to construct a xenograft tumor animal model. The weakly expressed circ_002136 gave rise to preventing the growth of solid HCC tumors in vivo, as evidenced by the reduction of tumor volume and weight (Fig. 9A-C). The above experimental results allow us to conclude that circ_002136 silencing can inhibit tumor progression in vivo.

## Discussion

HCC cells are highly susceptible to exhibiting uncontrollable malignant behaviors such as asymptomatic growth, long-distance migration and resistance to apoptosis [19]. During tumor development, exosomes-involved intercellular transfer of biologically active nucleic acid molecules and proteins can drive a shift in the tumor microenvironment and accelerate or inhibit local spread and multifocal growth of HCC [20, 21]. Emerging HCC studies have demonstrated that biological events are propagated between HCC cells with different characteristics by means of exosomes, affecting recipient cells growth, metastasis, etc. [22, 23] In the present study, we used exosomes of Huh7 and HA22T cell origins as the basis to reveal the effects of purified exosomes on the growth, metastasis and apoptosis of two types of recipient cells, respectively. The results of all three functional experiments confirmed that both exosome-stimulated Huh7 and HA22T cells exhibited the same changes in proliferation, migration, invasion, and apoptotic capacities, as evidenced by a soaring in cell viability, a promotion in cell motility, and an enhancement in cell resistance to apoptosis. The data obtained suggest that the functions of exosomes produced from Huh7 and HA22T may be mainly focused on influencing HCC tumorigenicity.

The essence of tumorigenesis is the transformation of normal cells to tumor cells. And the transport of differential genes in tumors by exosomes is one of the key drivers of oncogenic transformation and the dissemination of malignant biological behaviors. Recently, circRNA stored in exosomes of HCC cells has attracted increasing attention. Exosomal circUHRF1 [24], exosomal circPTGR1 [22], exosomal circRNA-100,338 [17], and exosomal circ_MMP2 [25] occupy a linchpin position in mediating HCC cells communication as well as assisting cancer metastasis. Circ_002136 was first identified in glioma, where it primarily performs oncogene functions that activate glioma cell growth and migration [16]. In the present study, we first found that circ_002136 had a similar distribution pattern in both HCC cell lines, being enriched in intracellular and exosomal compartments. Subsequently, our results confirmed that the ablation of circ_002136 could prevent cell growth and metastasis in vitro to some extent and confer elevated apoptotic capacity to the cells. However, the addition of exosomes counter-regulated these results. These data further support our conjecture that circ_002136 can be loaded inside exosomes to re-internalize into tumor cells and interfere with their biological effects.

Multiple circRNAs have been identified that can uptake and sequester key miRNA molecules associated with oncogenic transformation to change cancer progression. In this study, we demonstrated the inhibitory effect of circ_002136 on miR-19a-3p activity. In investigating the role of circ_002136/miR-19a-3p axis in HCC progression, we performed purposefully rescue experiments. The experimental data demonstrated that circ_002136 and miR-19a-3p co-mutilation can partially revert cellular functions and partially reverse the effect of circ_002136 on HCC progression. Indeed, the low expression pattern of miR-19a-3p in HCC tissues and cells supported its function as an oncogenic factor to impede the malignant behavior of cancer [26]. Extensive studies have also long established that miR-19a-3p was related to various cancer phenotypes, commonly containing cancer metastasis and drug resistance [27–30]. Yu et al. illustrated that overexpressed miR-19a-3p interferes with colorectal cancer metastasis by blocking EMT-related pathway activation via FOXF2 [28]. Feng et al. also linked miR-19a-3p expression to prostate cancer and identified its oncogenic role [29]. More importantly, high levels of miR-19a-3p can also be an independent prognostic factor for cancer patients [30]. Data-wise, the present study broadens the understanding of miR-19a-3p as an oncogenic factor took part in cancer progression.

MiRNAs function as active regulatory elements that actively weaken the stability of target mRNAs or manipulate their translation. MiR-19a-3p frequently targets different genes in multiple types of cancers, presenting an intricate network regulatory pattern. In this study, RAB1A was identified as a downstream gene monitored by miR-19a-3p. RAB1A belongs to the Rab1 protein isoform in the Rab protein family, which is an essential vesicle transporter between the endoplasmic reticulum and Golgi apparatus [31]. In cancer development, abnormal expression of RAB1A is involved in the progression of various cancers, such as colon cancer [32], tongue cancer [33], and glioma [34] in humans, all of which exhibit some degree of cancer-promoting effects in a gene dose-dependent manner. More notably, the promoting effect of RAB1A on malignant hepatocytes in HCC has also long been demonstrated. Consistent with previous results, in the present study, we found that the addition of RAB1A in the context of miR-19a-3p overexpression enhanced the malignant phenotype of HCC cells, suggesting that miR-19a-3p regulates tumor cell progression in a RAB1A-dependent manner. The regulatory network of exosome circ_002136 in HCC was further refined, i.e., circ_002136 affects the malignant transformation of HCC by means of miR-19a-3p/RAB1A axis.

More importantly, in the current study, we evaluated the clinical implications of circ_002136, miR-19a-3p, and RAB1A in HCC. We identified low expression of circ_002136 and RAB1A in early (stage I-II) HCC samples and gradually increased with disease progression. In contrast, the relative enrichment of miR-19a-3p in early (I-II) HCC specimens progressively decreased with disease progression. More notably, low expression of circ_002136 and RAB1A was associated with higher survival rates in HCC patients, whereas high miR-19a-3p expression effectively increased patient survival. These demonstrated that exosomal circ_002136, miR-19a-3p, and RAB1A were all promising prognostic indicators for HCC. Thus, our study extended the understanding of the function of circ_002136 and its downstream molecules in HCC.

## Conclusion

Overall, we illustrated that exosomes released from HCC cells could generate a remarkable enhancing effect on tumor cell activity, and fully demonstrated that the malignant biological behaviors of HCC cells could endow other receptor cells with higher activity and invasiveness under the help of exosomes. In addition, the upregulation of circ_002136 expression was found in HCC tissues and cells for the first time, and the circ_002136 level bore a close association with exosomes. The high abundance of exosomal circ_002136 targeted miR-19a-3p/RAB1A pathway in cancer cells to disrupt the stable microenvironment inside the tumor and exacerbate the malignant process of HCC tumors. In addition, circ_002136, miR-19a-3p and RAB1A were all related to the survival rate of HCC patients. These findings provide strong support for our insight into the mechanism of circRNA to be silenced as a therapeutic agent in HCC progression.

## Supplementary Information

**Additional file 1:** **Figure S1. **(A, B) Differential expression levels of ten circRNAs inexosome-treated/untreated Huh7 (A) and HA22T (B) cells were analyzed byqRT-PCR. (C, D) The knockdown efficiencies of circ_0005046 and circ_0008537 inHuh7 and HA22T cells were determined by qRT-PCR, respectively. Theproliferation and viability of Huh7 (C) and HA22T (D) cells after silencingcirc_0005046 and circ_0008537 were evaluated by CCK-8 assay. * *P*<0.05, ** *P*<0.01,*** *P*<0.001.

**Additional file 2:** **FigureS2. **Uncropped andunedited versions of the blots in Figure 1C.

## Data Availability

The data used to support the findings of this study are available from the corresponding author upon request.

## Abbreviations

circRNAs: Circular RNAs
HCC: hepatocellular carcinoma
qRT-PCR: quantitative RT-PCR
PLC: Primary liver cancer
Exo: Exosomes
TEM: : transmission electron microscopy
NTA: nanoparticle tracking analysis
CCK-8: Cell Counting Kit-8
OD: optical density
TUNEL: Terminal deoxynucleotidyl transferase (TdT)-mediated dUTP nick-end labeling
