# Supplementary material for: Exosome-transmitted circ_002136 promotes hepatocellular carcinoma progression by miR-19a-3p/RAB1A pathway
Source: BMC Cancer. 2022 Dec 7;22:1284. doi: 10.1186/s12885-022-10367-z (PMC9730599; doi:10.1186/s12885-022-10367-z)
Supplement: Supplementary file 1 — Additional file 1: Figure S1. (A, B) Differential expression levels of ten circRNAs inexosome-treated/untreated Huh7 (A) and HA22T (B) cells were analyzed byqRT-PCR. (C, D) The knockdown efficiencies of circ_0005046 and circ_0008537 inHuh7 and HA22T cells were determined by qRT-PCR, respectively. Theproliferation and viability of Huh7 (C) and HA22T (D) cells after silencingcirc_0005046 and circ_0008537 were evaluated by CCK-8 assay. * P<0.05, ** P<0.01,*** P<0.001. [file 12885_2022_10367_MOESM1_ESM.docx]

**Additional file 1:**


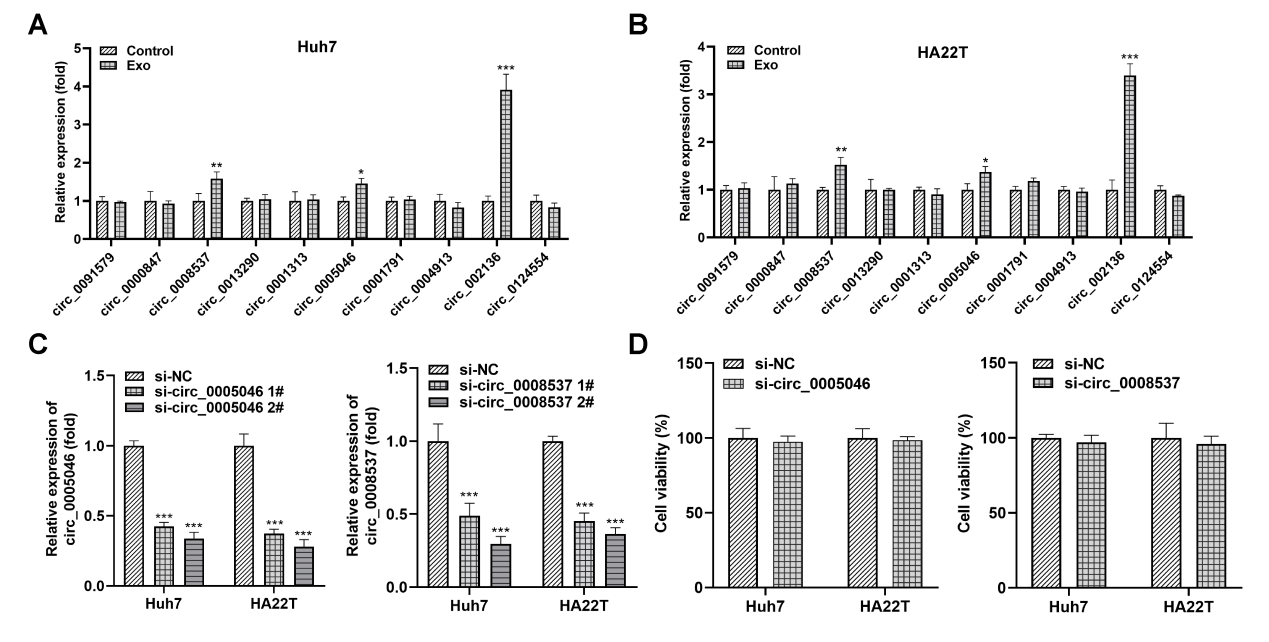


**Figure S1:** (A, B) Differential expression levels of ten circRNAs in exosome-treated/untreated Huh7 (A) and HA22T (B) cells were analyzed by qRT-PCR. (C, D) The knockdown efficiencies of circ_0005046 and circ_0008537 in Huh7 and HA22T cells were determined by qRT-PCR, respectively. The proliferation and viability of Huh7 (C) and HA22T (D) cells after silencing circ_0005046 and circ_0008537 were evaluated by CCK-8 assay. * *P*<0.05, ** *P*<0.01, *** *P*<0.001.
