# Supplementary figures and images for: Exosome-transmitted circ_002136 promotes hepatocellular carcinoma progression by miR-19a-3p/RAB1A pathway
Source: BMC Cancer. 2022 Dec 7;22:1284. doi: 10.1186/s12885-022-10367-z (PMC9730599; doi:10.1186/s12885-022-10367-z)

**Additional file 2:**

**(1)**


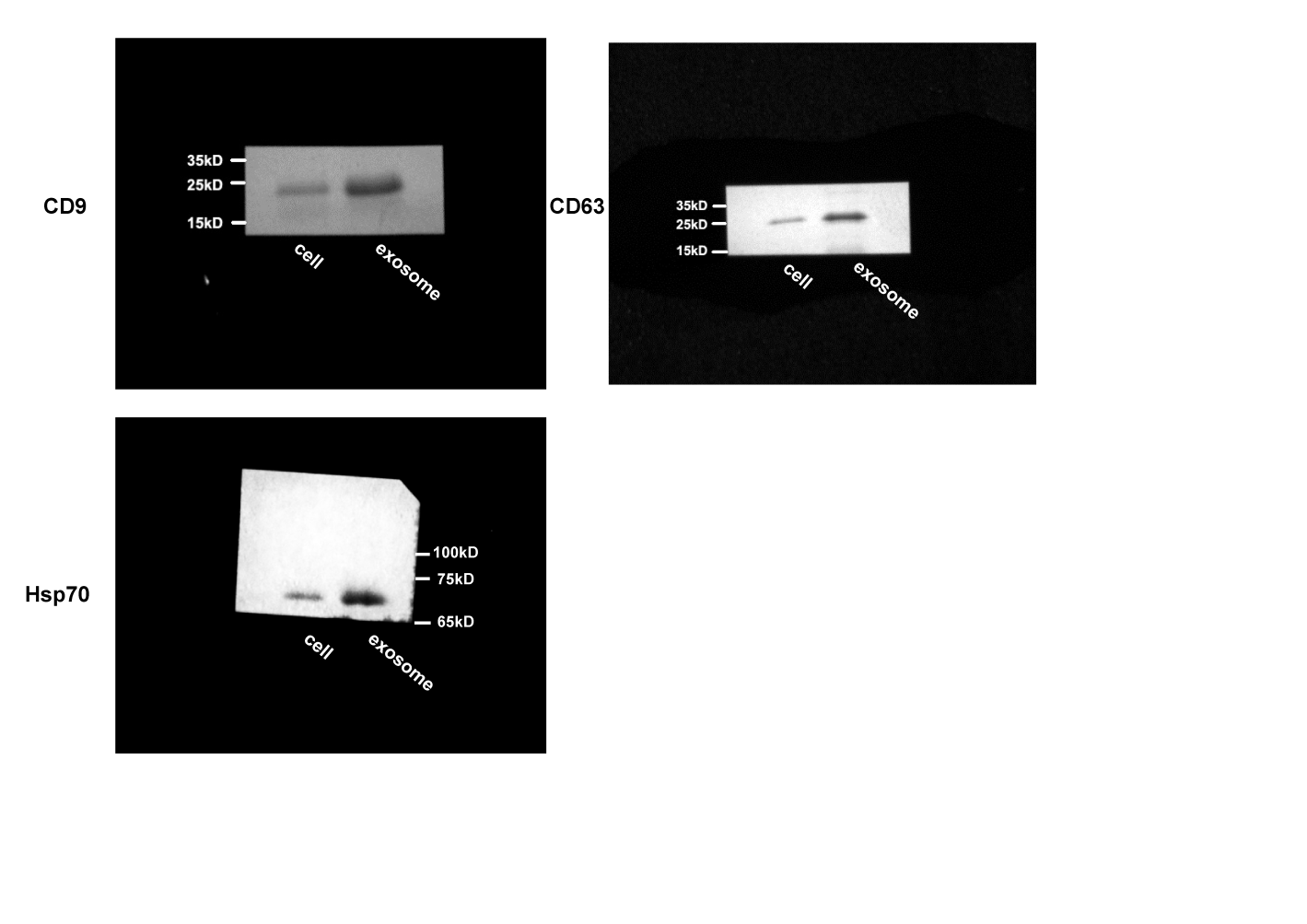


**(2)**


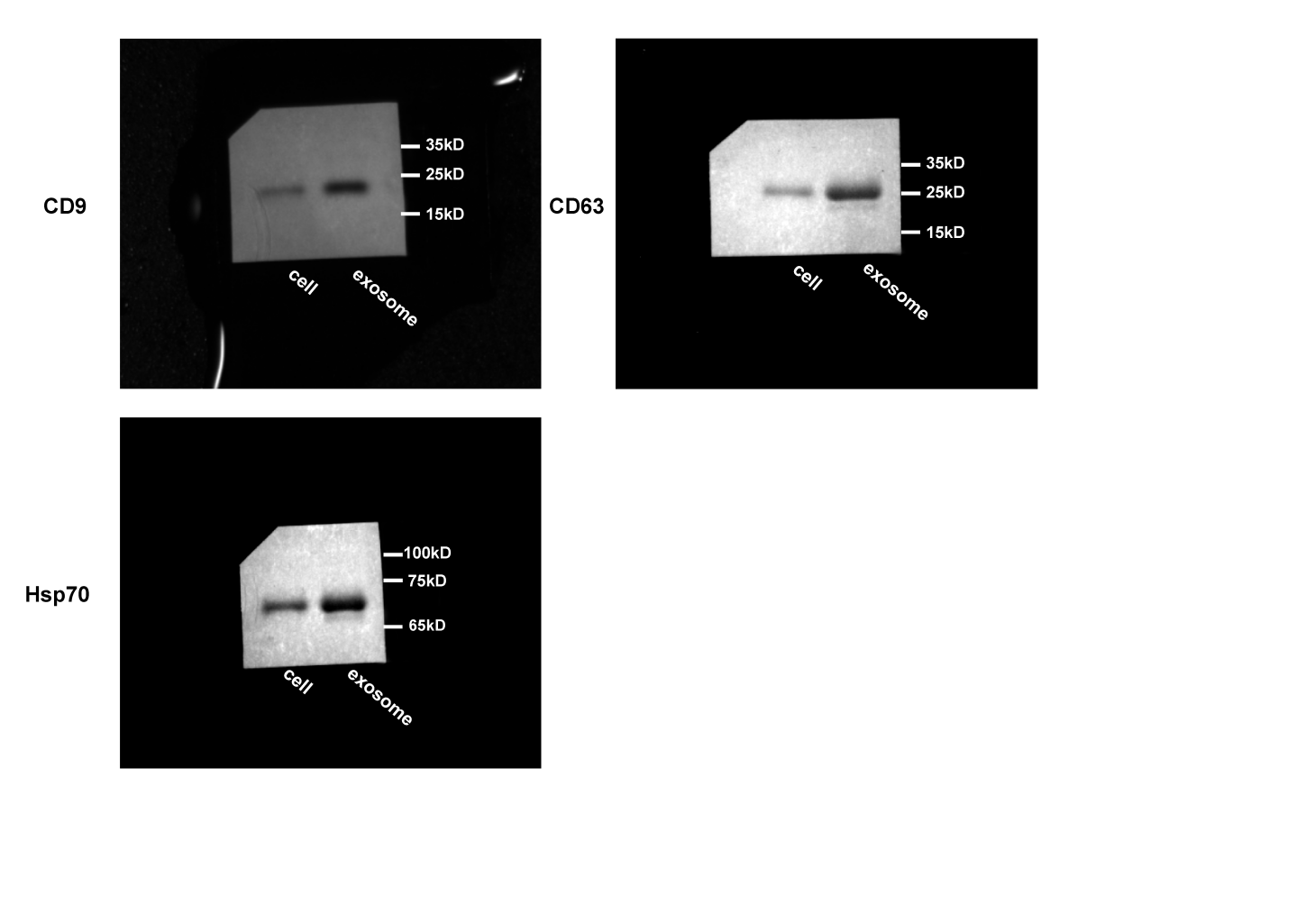


**(3)**


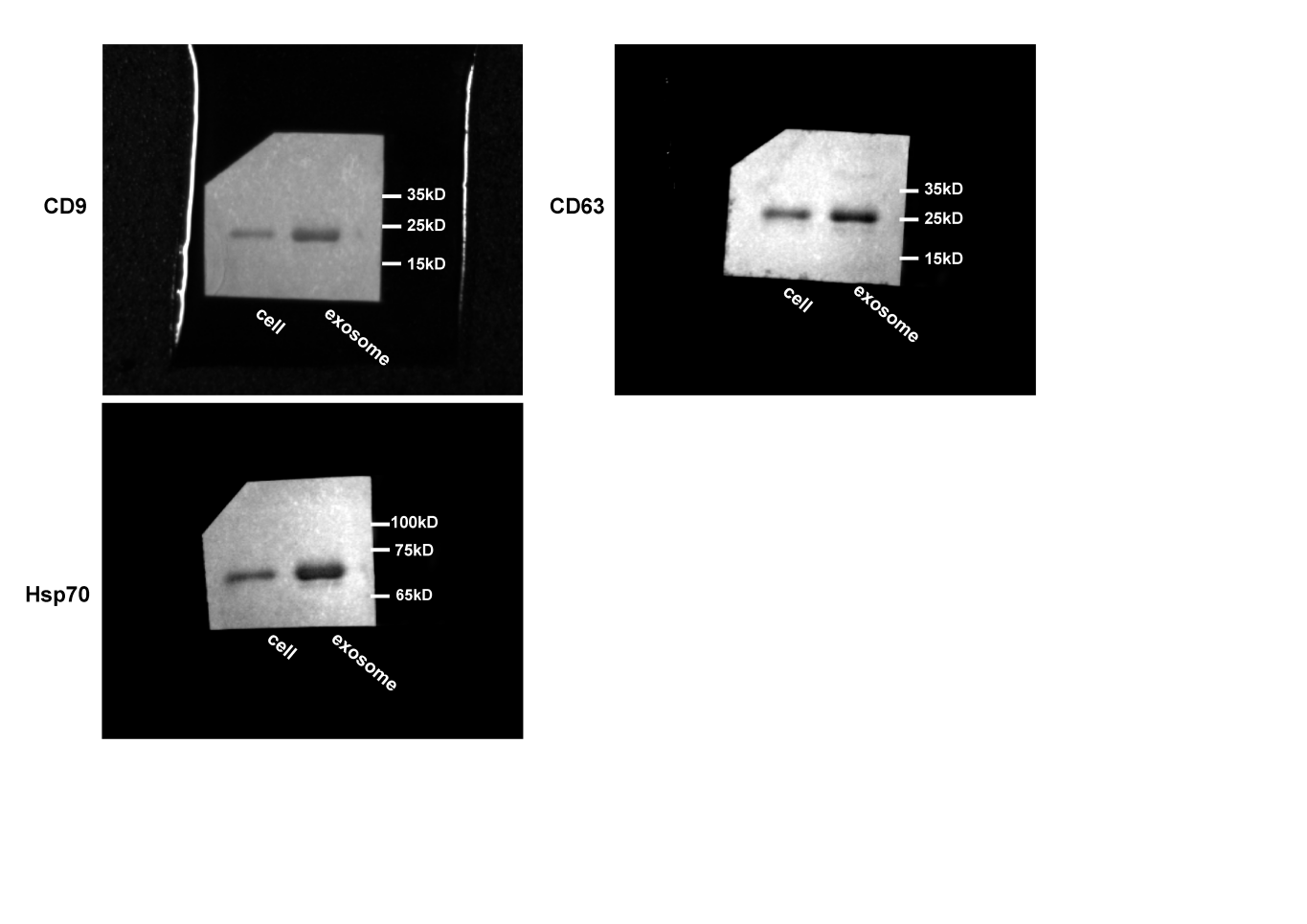


**Figure S2: Uncropped and unedited versions of the blots in Figure 1C.**

Supplement: Supplementary file 2 — Additional file 2: FigureS2. Uncropped andunedited versions of the blots in Figure 1C. [file 12885_2022_10367_MOESM2_ESM.docx]
